# Incidence of Velopharyngeal Insufficiency after Primary Cleft Palate Repair: A 27-Year Assessment of One Surgeon's Experience

**DOI:** 10.1055/a-2263-7857

**Published:** 2024-05-09

**Authors:** Chan Woo Jung, Hyung Joon Seo, Ye Seul Choi, Yong Chan Bae

**Affiliations:** 1 Department of Plastic and Reconstructive Surgery, School of Medicine, Busan National University, Busan, Korea; 2 Biomedical Research Institute, Busan National University Hospital, Busan, Korea; 3 Department of Rehabilitation Medicine, Busan National University Hospital, Busan, Korea

**Keywords:** cleft palate, velopharyngeal insufficiency, risk factor

## Abstract

**Background** Velopharyngeal insufficiency (VPI) is a major complication of cleft palate repair. The purpose of this study was to evaluate the incidence and predictive factors of VPI after cleft palate repair based on 27 years of one surgeon's experience.

**Methods** Medical records were retrospectively reviewed for 652 patients who underwent cleft palate repair between 1995 and 2021. After exclusion of those with other syndromes or developmental disorders, the study included 374 patients with sufficient follow-up until the age of 4 years, when language evaluation was possible. VPI status was categorized through subjective and objective tests into normal, VPI, and borderline. We analyzed potential differences in VPI incidence by multiple factors. Factors with significance were analyzed to confirm the relationships between subvariables.

**Results** Of the 374 patients, 311 (83.2%) exhibited normal pronunciation, 51 (13.6%) had VPI, and 12 (3.2%) were borderline. Primary cleft palate repair performed after 18 months was associated with a higher incidence of VPI than repair conducted before 18 months ( *p* = 0.005). The incidence of VPI was higher in cases of submucous cleft palate than in the other types based on the Veau classification ( *p* = 0.011). However, in the multivariable analysis, only the submucous type showed statistically significant results ( *p* = 0.026).

**Conclusion** A total of 374 people underwent primary cleft palate repair, and 13.6% of those with VPI required secondary therapy. The incidence of VPI was relatively high among patients with primary cleft palate repair after 18 months and patients with submucous cleft palate.

## Introduction

Cleft lip and palate is the world's most common congenital anomaly in the head and neck, affecting approximately 1 in 700 people in the United States and 1 in 500 in Korea. 1 2

Patients with cleft palate may experience issues such as feeding problems, aesthetic differences, hearing loss, malocclusion, and airway obstruction. 3 For this reason, the main purpose of primary cleft palate repair is to restore the proper function of the anatomical structures. 4 The goal of the repair is to extend the length of the soft palate, divide the nasal cavity and oral cavity, and recover the normal velopharyngeal function through repairing the sling of the levator muscle. 3 5

Postoperative velopharyngeal insufficiency (VPI) is characterized by postoperative hypernasality, nasal air emission, and compensatory articulation errors. 5 These characteristics impact the ability to speak and affect mental health, so patients with cleft palate tend to have low self-esteem and exhibit introversion. 4

In this way, VPI, which commonly affects patients after surgery, can be a measure of the success of primary palatal therapy. 6 One study indicated that the proportion of patients requiring secondary therapy due to VPI was usually 10 to 30%, 7 while others have demonstrated rates of 7.4 to 37.1%, representing a diverse range. 8 9 10 11 12 Although conditions vary from study to study, researchers have argued that VPI incidence is associated with various predictive factors, such as cleft palate type, surgical technique, age at primary cleft palate repair, surgeon experience, sex, and presence of postoperative palatal fistula. 13 14 15 16 17 18

The purpose of this study was to assess the incidence of postoperative VPI based on one surgeon's 27-year experience and to evaluate the predictive factors associated with VPI. For this evaluation, sex, age at primary cleft palate repair, presence of cleft lip, cleft palate type, cleft palate repair technique, and presence of postoperative palatal fistula were measured. The goal was to determine which patient factors increase the incidence of VPI after primary cleft palate repair by detecting the incidence of VPI and predictive factors.

## Methods

The Institutional Review Board of our institution approved this study (IRB No. H-2210-018-120). We retrospectively reviewed 652 consecutive primary palatal repair procedures performed at the XXXX University Hospital between 1995 and 2021 by a single surgeon. Of the 652 children, we excluded those with language delays due to other syndromes or developmental disorders (identified syndromes, nonsyndromic Robin sequence, or multiple congenital anomalies affecting the nervous system or head and neck). Of the remaining children, patients with sufficient medical records reaching 4 years of age, when language could be assessed, were selected. The incidence of VPI after primary cleft palate repair was investigated in the resulting 374 patients.

We assessed potential differences in VPI frequency based on factors such as sex, age at primary cleft palate repair, presence of cleft lip, cleft palate type, cleft palate repair technique, and presence of postoperative palatal fistula. In addition, statistical analysis was performed on factors which were statistically significant, to confirm the relationship between subvariables.

After the primary cleft palate repair, follow-up was performed twice a week until the second week, then every month until 3 months, then at 3-month intervals for up to 1 year, and then at 6-month intervals for ages of 4 to 5 years. Perceptual speech analysis, intraoral examination were performed between the ages of 4 and 5 years, when the language skills and sound production of the patients could be sufficiently assessed. An experienced cleft palate surgeon and a speech pathologist observed any nasal air emission, hypernasality, and compensatory misarticulation while the patients were speaking. 19 20 21 If the articulation is not accurate, the articulation was corrected through speech therapy and full speech evaluation was performed. In a comprehensive speech evaluation, pronunciation proficiency was assessed using the Pittsburgh Weighted Speech Scale and the Simple Speech Screening Protocol. Additionally, nasoendoscopy (NES) and video fluoroscopy (VFS) were conducted. 19 22 A proficient plastic surgeon meticulously examined the results of the pronunciation test, along with the NES and VFS images, while concurrently evaluating the closing pattern of the velopharyngeal sphincter. Velopharyngeal function was evaluated based on these tests and classified into normal, VPI, and borderline.

Patients capable of normal pronunciation were classified as normal. VPI included patients who required secondary surgery with severe hypernasal speech and audible nasal emission. Borderline included patients with mild hypernasal speech and mild nasal emission, for whom secondary surgery was not required. 23

The type of cleft palate was classified into five categories using the Veau classification system, along with the submucous type. These categories are as submucous cleft palate, Veau I (cleft soft palate), Veau II (hard/soft cleft palate), Veau III (unilateral cleft lip/palate), and Veau IV (bilateral cleft lip/palate). 18

### Surgical Technique

Primary cleft palate repair was performed at approximately 12 months of age. However, in some cases, diagnosis was delayed due to other medical issues. For cases of submucous cleft palate, repair was performed when the cleft palate surgeon detected a speech disorder during follow-up. 4 6

To maximize tissue mobilization and flap vascularity, the cleft palate repair technique was selected based on the extent of the cleft. In the early days when the operator started the cleft palate repair, cleft palate repair was performed mainly using the Veau–Wardill–Kilner techniques or intravelar veloplasty. At the same time, in cases of submucous cleft palate, cleft repair was performed using the Furlow double-opposing Z-plasty.

In most cases, repair was performed using the modified cleft palate repair method (Busan modification) for Veau classes I and II. 24 However, even in the case of Veau classes I and II, two flap repair was performed if the width of the cleft was wide or extended to the interior. For Veau classes III and IV, the surgeon performed two-flap repair, and a Vomer turnover flap was used to close the anterior nasal mucosa.

The modified cleft palate repair method (Busan modification) is a method of two-flap palatoplasty that does not involve a front V-shaped incision. It involves radical muscle dissection, repositioning, and a local relaxing incision. Eventually, the levator muscle is retrorepositioned, which is characterized by the reconstruction of the muscle sling through repair of the levator muscle. 24

### Statistical Analysis

Statistical Package for the Social Sciences (SPSS; ver. 22.0; SPSS Inc., Chicago, IL) was used for all statistical analysis. We conducted an investigation to ascertain whether variations in the incidence of VPI could be attributed to an array of factors, including sex, age at primary palatoplasty, the presence of cleft lip, cleft palate type, the specific technique employed for cleft palate repair, and the presence or absence of postoperative fistula formation. Statistical analysis was performed by summing VPI and borderline cases. The independent *t* -test, analysis of variance, Welch analysis, and Logistic regression (multivariable analysis) were performed on factors with significant values using IBM SPSS ver. 22 (IBM Corp., Armonk, NY), and the relationships between subvariables were confirmed. Statistical significance was considered to be indicated by *p* < 0.05.

## Results

Of the 374 patients, 311 (83.2%) exhibited normal pronunciation, while 63 (16.8%) showed abnormal pronunciation. Of the 63 patients with abnormal velopharyngeal function, 51 (13.6%) had VPI requiring secondary therapy, and 12 (3.2%) were borderline patients.

In univariate analysis, sex, presence of cleft lip, cleft palate repair technique, and presence of palatal fistula showed no statistically significant correlations with the incidence of VPI. In contrast, the age at cleft palate repair significantly predicted the incidence of VPI following cleft palate repair ( *p* = 0.005; Table 1 ). Regarding type of cleft palate, the incidence of VPI was higher in cases of submucous cleft palate (40.0%) than in the other types ( *p* < 0.001; Table 1 ).

**Table 1:** Comparison of the incidence of borderline and velopharyngeal insufficiency according to each factor (univariate analysis)

Factors	Number of patients	Number of abnormal	Incidence of abnormal (%)	*p* -Value
Patient sex				
Male	155	24	15.5	0.555
Female	219	39	17.8	
Age at cleft palate repair (months)				
9–12	232	32	13.8	0.005 a
13–18	66	7	10.6	
>18	76	24	31.6	
Associated cleft lip				
Yes	93	12	12.9	0.212
No	281	51	18.1	
Associated fistula				
Yes	12	5	41.7	0.114
No	362	58	16.0	
Extent of cleft				
Veau Class I	61	8	13.1	<0.001 a
Veau Class II	176	26	14.8	
Veau Class III	65	9	13.8	
Veau Class IV	27	2	7.4	
Submucous cleft palate 40.0	45	18	40.0	
Cleft palate repair technique				
Busan modification	183	33	18.0	0.114
Furlow double opposing Z-plasty	18	4	22.2	
Two-flap	127	23	18.1	
Veau–Wardill–Kilner	46	3	6.5	

Abnormal: velopharyngeal insufficiency + borderline.

a Significant value ( *p* < 0.05).

In our study, we conducted additional analyses using a multivariable approach to investigate the risk factors. In contrast to the univariate analysis, we did not obtain statistically significant results for age at cleft palate repair. Notably, when comparing submucous cleft palate to Veau class I as the reference, it exhibited a significantly higher incidence rate ( *p* = 0.026; Table 2 ).

**Table 2:** Multivariate logistic regression analyses for the incidence of velopharyngeal insufficiency (95% confidence interval)

Factor	OR [95% CI]	*p* -Value
Patient sex		
Male	Ref.	
Female	1.155 [0.635, 2.100]	0.638
Age at cleft palate repair (months)		
9–12	Ref.	
13–18	0.676 [0.278, 1.648]	0.390
>18	1.753 [0.771, 3.988]	0.180
Associated cleft lip		
Yes	Ref.	
No	1.351 [0.298, 6.134]	0.697
Associated fistula		
Yes	Ref.	
No	3.091 [0.846, 11.287]	0.088
Extent of cleft		
Veau Class I	Ref.	
Veau Class II	0.970 [0.382, 2.465]	0.949
Veau Class III	0.582 [0.092, 3.686]	0.565
Veau Class IV	0.258 [0.027, 2.463]	0.239
Submucous cleft palate	3.486 [1.164, 10.441]	0.026 a
Cleft palate repair technique		
Busan modification	Ref.	
Furlow double opposing Z-plasty	0.469 [0.127, 1.724]	0.254
Two-flap	2.093 [0.918, 4.768]	0.079
Veau-Wardill-Kilner	0.559 [0.148, 2.110]	0.391

Abbreviation: CI, confidence interval; OR, odds ratio.

Odds ratio is the ratio of the odds of failure for the second procedure to that of the first. Values above 1.00 favor the first, whereas values below 1.00 favor the second.

a Significant values ( *p* < 0.05).

## Discussion

VPI refers to nasal air emission and/or hypernasality during speech due to mechanical restriction, malposition, or reciprocity of velar tissue. 5 However, the criteria for defining borderline VPI and requirement of secondary surgery due to VPI differ among studies. In some papers, borderline VPI was assessed by the degree of bubbling or gap using only NES, 25 while other authors have used speech intelligibility criteria such as hypernasal resistance, nasal air emission, and intraoral pressure. 8 23 In this study, 13.6% of patients required secondary surgery due to VPI, and 83.2% of patients showed competent velopharyngeal function. In previous studies, the proportion of VPI patients who needed secondary surgery after primary cleft repair has ranged from 7.4 to 37.1%, and rates of competent velopharyngeal function have ranged from 85.1 to 72.4%. 8 9 10 11 12 However, as mentioned above, a simple comparison is difficult because every study differs in their criteria for recommendation of secondary surgery, borderline VPI, palatoplasty technique, age at palatoplasty, and patient exclusion.

In this study, the proportion of patients requiring secondary surgery due to VPI was 13.6%. Among the patients who underwent primary cleft palate repair between 1995 and 2018, 222 patients discontinued follow-up. In Korea, the coverage of National Health Insurance was 97.2% of the population in 2018, while 2.8% was covered by Medical Aid Program. In addition, in 2020, each Korean citizen received 14.7 outpatient treatments per year, the highest among Organization for Economic Co-operation and Development (OECD) countries. 26 For this reason, if parents think their child has a problem after a cleft palate repair, they are likely to actively visit the outpatient clinic. Conversely, if any were considered to have a problem after palate repair, they very likely would have received appropriate treatment through continuous follow-up. Therefore, the incidence of VPI would likely be lower if follow-up was conducted until all patients could undergo a full speech evaluation.

In a study by Jackson et al, 8 which compared many patients over a long duration as in the present study, the proportion of patients requiring secondary therapy was lower than in this study (8.1 vs. 13.6%). The rate of those showing comprehensive velopharyngeal function was also higher in this study (72.5 vs. 83.2%). However, the proportion of patients with borderline velopharyngeal function differed; Jackson et al 8 gave the proportion of borderline patients as 21.7%, while this study indicated an incidence of 3.2%. Differences in the inclusion criteria used to define borderline velopharyngeal function may be a factor explaining this difference.

The patients with submucous cleft palate underwent primary cleft palate repair at an average of 44 months, and the incidence of VPI among them was 40.0%, which was significantly higher than among patients with other types. Furthermore, in multivariable analysis, this was the sole risk factor that maintained statistical significance ( *p* = 0.026). Submucous cleft palate is one subset of cleft palate, and the diagnosis period tends to be later than for other types. 27 28 Many patients exhibit no symptoms at the time of diagnosis, and normal pronunciation may appear later. 28 For this reason, even after diagnosis, close observation is performed instead of immediate surgery. If a problem with velopharyngeal function is revealed during this observation by performing full speech evaluation around the age of 4, surgery is considered. Thus, surgery is delayed relative to other types of cleft palate.

The optimal age for cleft palate repair should be determined in consideration of speech outcomes and reduced maxillofacial growth distance. In this hospital, we recommend performing cleft palate repair at 10 to 11 months, but surgery was performed after 18 months when the diagnosis of the patient was delayed by other medical issues. The average age at cleft palate repair in the group that underwent surgery after 18 months was approximately 41 months. Most studies have indicated that the later the primary cleft palate repair, the higher the VPI incidence rate. 12 15 16 29 In this study, a discrepancy in the results emerged between univariate analysis and multivariable analysis concerning primary cleft palate repair. In univariate analysis, it was observed that the incidence of VPI increases when cleft palate repair is performed beyond 18 months. However, in multivariable analysis, although VPI incidence displayed an increase, the results did not attain statistical significance ( *p* = 0.170). This finding is believed to be associated with the surgical timing for submucous type infants, as mentioned earlier. Submucous type infants, as mentioned before, who are deemed to have a higher likelihood of VPI, had an average diagnostic age of 44 months. Most submucous type infants fell into the group of those undergoing surgery beyond 18 months, and univariate analysis yielded statistically significant results ( *p* = 0.005). Nonetheless, in multivariable analysis, while the odds ratio was higher for the 9 to 12 months group, the results did not achieve statistical significance.

We distinguished clefts by type into the submucous type and Veau classification levels. Some previous studies have suggested a higher incidence of VPI with increasing Veau classification, 13 14 18 while another argued that no correlation exists between Veau hierarchy and VPI incidence. 25 In this study, the incidence rate was approximately 13 to 14% in Veau classes I to III, and 7% in Veau class IV, but the difference was not statistically significant ( Table 2 ). The incidence of VPI did not show a statistically significant relationship with the presence or absence of cleft lip, but when cleft lip was present, the incidence was lower than when it was absent (12.9 vs. 18.1%). If a cleft lip is accompanied by a cleft palate, the classification is highly likely to be Veau class III or IV 5 ; thus, this result aligned with the finding of no significant association between Veau class and VPI incidence.

In this study, VPI incidence was also investigated in relation to sex. The incidence was 15.5% in boys and 17.8% in girls, which did not constitute a statistically significant difference ( *p* = 0.555). Some previous studies have alternatively reported a difference in VPI incidence between boys and girls, 6 with higher incidence in boys. 30 However, our results indicated that sex did not affect VPI incidence rate, which was consistent with Yang et al. 31

We evaluated whether the presence of palatal fistula and the incidence of VPI were related. Previous studies have indicated that fistula affects the development of VPI. 16 In our study, patients with palatal fistula had a higher incidence and higher odds ratio of VPI than patients without palatal fistula, although this result was not statistically significant given the group size.

In our study, no correlation was found between surgical technique and the incidence of VPI ( *p* = 0.242). However, previous studies have introduced the double-opposing Z-plasty as a technique that can reduce the occurrence of VPI. 32 33 34 We compared the four types of surgical methods using Welch and multiple regression analyses. The results indicated a difference in VPI frequency by technique, but it was not statistically significant. The author initially used the Furlow double-opposing Z-plasty technique to repair submucous cleft palate, but this was gradually discontinued due to a high incidence of fistula. For this reason, fewer patients were operated on using a Furlow double-opposing Z-plasty technique ( *n* = 18) than a Busan modification technique ( *n* = 183) or a two-flap technique ( *n* = 127), which indicates a lack of experience in the Furlow double-opposing Z-plasty technique. We believe that the experience levels of the technician with each surgical method may have influenced the incidence of VPI.

The main advantage of this study is relatively large scale within a single craniofacial center that allow for comparison of palate repair in several groups. But, this study has limitations due to it being a retrospective analysis. Additionally, while our institution's recording method was transferred from manual to electronic, there is a possibility that omissions occurred in patient information and test results. In addition, given the inherent deficiencies of retrospective studies, there was no means to ensure exact equivalence in all demographics, cleft palate types, and surgeries performed. Although we tried to control for various confounding factors, the author's preferred surgical method cannot be ignored. In future studies, these issues should be carefully considered.

In summary, in this study, VPI requiring secondary surgery was found in 13.6% of patients and competent velopharyngeal function in 83.2%. Submucous type was only predictors of VPI incidence. The incidence of VPI did not show a statistically significant relationship with the Veau classification level and cleft palate repair technique. The surgical method is selected based on the surgeon's preference, but as the surgeon's experience with the technique increases, the surgical outcome can be improved.
